# Direct *Streptococcus pneumoniae* real-time PCR serotyping from pediatric parapneumonic effusions

**DOI:** 10.1186/1471-2431-14-189

**Published:** 2014-07-24

**Authors:** Robert Slinger, Lucie Hyde, Ioana Moldovan, Francis Chan, Jeffrey M Pernica

**Affiliations:** 1 Department of Laboratory Medicine and Pathology and Department of Pediatrics, University of Ottawa, Ottawa, ON, Canada; 2 Children’s Hospital of Eastern Ontario, Ottawa, ON, Canada; 3 Department of Pediatrics, McMaster University, Hamilton, ON, Canada

**Keywords:** Real-time PCR, Empyema, *Streptococcus pneumoniae*, Serotype, Vaccine

## Abstract

**Background:**

To determine the serotypes of *Streptococcus pneumoniae* responsible for pneumonia complicated by parapneumonic effusion in children, we performed real-time PCR based pneumococcal “serotyping” directly on parapneumonic fluid samples.

**Methods:**

Specimens were collected at two children’s hospitals in Ontario, Canada from 2009 to 2011. Samples in which *S. pneumoniae* was detected by PCR were tested with serotype-specific 5′exonuclease PCR assays for the 13 serotypes contained in the 13-serotype pneumococcal vaccine.

**Results:**

Thirty-five *S. pneumoniae* PCR-positive pleural samples were studied. Pneumococcal serotyping PCR assays were positive for 34 of 35 (97%). Serotype 3 was detected most frequently, in 19/35 (54%), followed by serotype 19A in 9/35 (26%), serotype 7 F/A in 4/35 (11%), serotype 1 in 1/35 (3%), and serotype 6A also in 1/35 (3%).

**Conclusions:**

PCR testing demonstrated that the vast majority (97%) of *S. pneumoniae* parapneumonic effusions were caused by serotypes present in the 13-serotype vaccine that were not present in the original 7 serotype vaccine. This suggests that use of the 13-serotype vaccine could potentially prevent many *S. pneumoniae* pneumonias complicated by parapneumonic effusion in our region, provided serotype replacement does not occur.

## Background

Pneumonia complicated by parapneumonic effusion is a severe infection in children that leads to prolonged hospital stay and risk of intensive care unit admission, as well as other morbidities [1]. The incidence of pneumonia complicated by parapneumonic effusion or empyema in children has increased over the past 10-15 years in many areas of the world, including the US, Canada, Europe, Asia, and Australia [2-7]. This increase occurred despite the introduction of a 7-serotype conjugated *Streptococcus pneumoniae* vaccine (pneumococcal conjugated vaccine, 7-serotype, PCV7) for routine childhood immunization in many of these regions; in some studies, the spike in parapneumonic effusion incidence appeared to occur subsequent to the widespread use of PCV7 [3,5], whereas in other regions the increase was documented prior to universal pneumococcal vaccination [2,6-8]. As *S. pneumoniae* is the major cause of community-acquired pneumonia in adults and children, PCV7 vaccination programs led to dramatic decreases in the number of children presenting with and admitted for pneumonia; however, the increase in parapneumonic effusion rates was unexpected.

Two main hypotheses were generated to explain this phenomenon. Some investigators believed that the empyema increase was observed due to a proportional rise in bacterial pneumonia due to bacteria other than *S. pneumoniae*. Grijalva et al., for example, documented a four-fold rise in staphylococcal empyema in American infants between 1996-98 and 2005-07 [2]; however, it should be noted that they did not find corresponding increases in empyema caused by *S. aureus* in children aged 2-4 years or in all pediatric patients aged < 18 years [9].

The other plausible explanation was that the excess parapneumonic effusions resulted from a rise in *S. pneumoniae* infections due to serotypes not present in the PCV7, referred to as ‘serotype replacement’. Many investigators have shown an association between parapneumonic effusion/empyema and certain pneumococcal serotypes both after the introduction of PCV7, including 1, 3, 19A, and others [7,10-13].

In an earlier study of molecular diagnosis of pneumonia complicated by parapneumonic effusion in children and youth from two tertiary care pediatric hospital sites in Ontario, Canada, we performed real-time multiple uniplex PCR for numerous bacterial pathogens on pleural fluid samples from 56 different children [14]. A bacterial pathogen was detected by PCR in 46/56 (82%) of the pleural fluids. *S. pneumoniae* was the most commonly detected bacterium by PCR, found in 35/56 (63%) of the pleural samples; in contrast, only 8/56 (14%) children had *S. pneumoniae* detected by culture of blood or pleural fluid. No samples were positive for *S. aureus* by culture or PCR. These results suggested that *S. pneumoniae* was responsible for most of the pneumonias complicated by parapneumonic effusion at our hospitals.

The objective of this current study was to determine which serotypes of *S. pneumoniae* were responsible for these infections, and whether the responsible serotypes were included in the newer 13-serotype pneumococcal conjugated vaccine (PCV13) vaccine and thus might be preventable. Of note, in Ontario, Canada, where we performed this study, the 7-serotype pneumococcal vaccine was introduced in 2005. A 10-serotype pneumococcal vaccine (Synflorix®) replaced PCV7 in October 2009, and was itself replaced by PCV13 (Prevnar13®) in November 2010.

As described below, we performed direct molecular “serotyping” on *S. pneumoniae* PCR-positive pleural fluid samples. We used real-time PCR assays for the 7 serotypes included in PCV7 as well as assays for the 6 additional serotypes present in PCV13.

## Methods

Ethics approval was obtained from the Children’s Hospital of Eastern Ontario and the McMaster Research Ethics Review Boards for study of anonymized residual pleural fluid samples that would otherwise have been discarded. Pleural and blood culture results were recorded for comparisons to PCR results, but clinical patient information such as age and immunization status was not recorded. Pleural fluid samples were collected at two children’s hospitals in Ontario, Canada, the Children’s Hospital of Eastern Ontario and the McMaster Children's Hospital, from 2009 to 2011. Two of the investigators (RS and JP) reviewed microbiology laboratory specimen records for samples submitted for pleural fluid culture and admission records for children hospitalized with a diagnosis of pneumonia and/or parapneumonic effusion. Pleural fluids from patients without a diagnosis of pneumonia were not eligible for inclusion.

Thirty-five *S. pneumoniae* PCR- positive samples collected as part of the earlier study [14] were available for PCR serotyping. *S. pneumoniae* was grown in culture from only 3 of these 35 pleural fluids, likely because most patients had received antibiotic therapy prior to pleural fluid specimen collection. Blood cultures were positive for *S. pneumoniae* in five additional patients. Pleural samples had been stored initially in sterile containers at -80°C; DNA was later extracted with an automated device (iPrep, Life Technologies, Carlsbad, CA), and nucleic acid extracts were also stored at -80°C.

Pneumococcal PCR serotyping assays for the 13 serotypes contained within PCV13 (1, 3, 4, 5, 6A, 6B, 7 F, 9 V, 14, 18C, 19A, 19 F, and 23 F) were performed on these 35 specimens. The sequences of the primers and probes used for detection of each serotype are shown in an additional file [see “*Streptococcus pneumoniae* real-time PCR molecular serotyping assays used with pleural fluid specimens”, Additional file 1: Table S1] [15,16]. Serotype-specific assays were selected for use whenever possible but PCR assays capable of detecting only the vaccine serotype were not available for some serotypes. For example, the assay used to detect the 7 F vaccine serotype also detects the related 7 F non-vaccine serotype.

Real-time singleplex PCR was performed for each serotype assay. PCR assays (5′ exonuclease probe type) were obtained from Life Technologies Inc. PCR reaction assays were prepared in 10 μL volumes in 96-well PCR plate. TaqMan Fast Advanced Master Mix (Life Technologies) was used for amplification. A negative control (no template) was performed with each sample. PCR plates were covered with MicroAmp® Optical Adhesive Film (Life Technologies) to prevent cross-contamination. PCR was performed with a 96 well fast cycling block on a ViiA7 thermocyler (Life Technologies) using 40 cycles of 2-temperature thermocyling (95°C x 15 seconds and 60°C × 30 seconds).

Positive controls were performed for all assays using DNA extracted from cultures of *S. pneumoniae* of known serotypes typed at a national reference laboratory, or, if cultured isolates for a certain serotype were not available, DNA oligonucleotides that were identical to the target sequences were purchased (Integrated DNA Technologies, Inc., Coralville, Iowa) and used as positive controls. Negative (no template) controls were run with each PCR to ensure cross-contamination had not occurred. Threshold cycle (Ct) values were obtained, with Ct values <35 considered to be positive.

For analysis, positive serotype results by PCR were considered to be PCV13-preventable if they were positive with one of the 9 vaccine serotype-specific assays, as possibly vaccine preventable if they were positive with one of the 4 assays that detected more than one serotype, and as non-vaccine preventable if they were negative with all 13 PCR assays.

## Results

**Figure 1:**
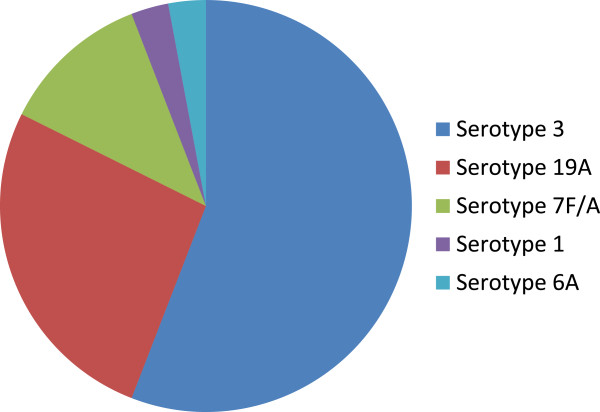
***S. pneumoniae *serotypes by direct real-time PCR typing of pediatric pleural fluid specimens.** The five serotypes detected are included in the newer 13-serotype conjugated *S. pneumoniae* vaccine but were not present in original 7-serotype vaccine.

Thirty-five *S. pneumoniae* PCR- positive samples were studied: 13 specimens from 2009, 9 specimens from 2010, and 13 specimens from 2011. Molecular serotyping detected one of the PCV13 serotypes in 34 of the 35 (97%) *S. pneumoniae* PCR- positive samples. As shown in Figure 1, serotype 3 was most frequently detected, present in nineteen specimens (54%), followed by serotypes 19A in nine specimens (26%), serotype 7 F/7A in four specimens (11%), serotype 1 in one specimen (3%), and serotype 6A also in one specimen (3%).

Of note, traditional serotyping results were available for four of the eight cases in which *S. pneumoniae* had been grown from either pleural fluid or blood culture, and in all four culture-positive samples results were concordant with the PCR serotyping results (these were two positive for serotype 19A, one for serotype 3, and one for serotype 7 F).

## Discussion

Thirty-four of the 35 *S. pneumoniae* PCR-positive parapneumonic fluid samples could be serotyped using PCR assays for serotypes (3, 19A, 1, 6A, 7 F/A) included in PCV13. Thirty of these thirty-four were considered definitely PCV13-preventable infections, since the PCR assays used were specific for one serotype. Four samples were positive with a PCR assay that detects both serotypes 7 F and 7A, so were classified as having been possibly PCV13-preventable infections (since serotype 7 F but not 7A is included in PCV13). One PCR-positive pleural fluid was negative with all 13 PCR assays and was classified as not PCV13-preventable.

In contrast to some other studies in which serotype 1 infections have been most common [2,12,13,17], we found only one case of empyema caused by *S. pneumoniae* serotype 1. The majority of parapneumonic effusions in our study were found to be caused by serotype 3, which has recently been reported to be associated with empyema even in children immunized with PCV13 in Greece [10]. Serotype 19A was the second most frequently detected serotype by PCR in this study. This serotype was reported as the most common cause of culture-positive invasive *S. pneumoniae* infections in all age groups in Province of Ontario in 2009 [18].

Notably, the five serotypes detected by PCR (assuming the serotype 7 detected was indeed 7 F and not 7A) were all among the six serotypes added to PCV13 that were not present in the original PCV7. This is similar to studies in other countries in the post-PCV7 era. For example, in an Australian study conducted in 2008-9, 90% of the pneumococcal serotypes detected in pleural fluid samples were among the 6 new serotypes in PCV13, with serotype 19A (41%) and 3 (31%) found most commonly [19]. Routine PCV7 use began in Australia in 2005, but PCV10 or PCV13 had not been introduced at the time of this study. In an American study performed in 2009, 77% of *S. pneumoniae* PCR-positive pleural fluid samples were due to the 6 new serotypes; serotype 7 F/A was most frequently detected (47%), while serotypes 19A and 3 were both detected in 13% of samples [20]. This study was also conducted after the introduction of PCV7 but prior to use of PCV13.

This study also demonstrated the usefulness of PCR methods for identification of *S. pneumoniae* serotypes in children with parapneumonic effusions, since cultures are positive for *S. pneumoniae* in only a minority of patients of these patients. Our finding that *S. pneumoniae* was detected by culture (of blood or pleural fluid) in only 23% (8/35) of children with PCR-positive pleural fluid samples is consistent with other reports [17,18]. This suggests that molecular methods such as PCR are needed to help guide surveillance and vaccination programs.

A retrospective review of children admitted to one of the study site hospitals (the Children’s Hospital of Eastern Ontario) in 2007- 2008, provides some relevant information about the epidemiology of pneumonia and effusion in our region just prior to the start of the *S. pneumoniae* serotyping PCR study [1]. In the review, 135 children were found to have been hospitalized with pneumonia over the two year period. The mean age at admission was 4.8 years (range 0-17 years), and 41% had received antibiotics prior to admission. With regard to parapneumonic effusions in the study period, twenty-two children had pleural fluid cultures, or on average 11/year, and two of 22 (9.1%) samples were culture- positive. The median length of stay was longer for those with pleural effusions (9 days) than for those without effusions (3 days), and presence of pleural effusion was associated with admission to the intensive care unit.

During the PCR serotyping study years, the number of parapneumonic specimens obtained at this site was 22 in 2009, 13 in 2010, and 12 in 2011. Thus, for unknown reasons, an increase in pleural specimens cultured was seen in 2009, which then appears to have decreased to numbers similar to the 2007 and 2008 years.

Our PCR serotyping study has several limitations that should be noted. From a technical viewpoint, the PCR assay used for serotype 7 detected both the 7 F vaccine serotype and the 7A non-vaccine serotype, making it uncertain as to whether infections that were PCR-positive with the assay could be prevented with the PCV13 vaccine. Unfortunately, no other PCR assay specific for only 7 F could be found in the medical literature.

Another limitation is that since the pleural fluids assayed were obtained from children with parapneumonic effusions at two children’s hospitals in the Province of Ontario, Canada, we cannot necessarily generalize our findings to the entire Provincial population. However, it is reasonable to hypothesize that our findings may reflect overall trends in our Province.

An additional limitation is that we are due to the anonymous nature of the specimens tested we are not aware of the age or *S. pneumoniae* vaccine status of the children whose samples were studied. Since the PCV7 was introduced in 2005 in our Province, it is likely that some of the children in this study had received the PCV7 vaccine.

Finally, the number of *S. pneumoniae-*PCR positive samples that were available for PCR serotyping (35) was relatively small. However, this number of samples is comparable to those obtained in similar studies from the USA (45 samples) and Australia (43 samples), and reflects the fact that pneumonia complicated by parapneumonic effusion is a relatively infrequent infection. As well, despite the relatively small numbers, we observed a dramatic difference in the number of cases for which serotyping could be performed using PCR as compared to culture. PCR enabled *S. pneumoniae* serotyping to be performed for approximately four times more infections (35 patients vs. 8 patients) than was possible with culture.

## Conclusions

In summary, our study suggests that many cases of pneumococcal pneumonia with empyema that occurred between 2009 and 2011 in our region might have been prevented by PCV13 vaccination. We may therefore observe a decrease in the incidence of pneumonia complicated by parapneumonic effusion in future now that PCV13 has been integrated into the Provincial universal immunization program. However, serotype replacement with new serotypes not in PCV13 is also a real possibility.

Continued surveillance using molecular serotyping methods such as real-time PCR is probably the most effective tool to monitor which *S. pneumoniae* serotypes are responsible for pneumonias complicated by parapneumonic effusion. This will allow the effectiveness of PCV13 and the need for future development of new pneumococcal vaccines to be assessed using much more comprehensive information than is currently available using culture-based surveillance. Molecular surveillance will also be important to determine whether or not *S. pneumoniae* serotype 3 pneumonias with parapneumonic effusion are effectively prevented by the use of PCV13, as this serotype has caused infection in some patients immunized with PCV13 [10].

## Abbreviations

PCR: Polymerase chain reaction; PCV7: 7 serotype pneumococcal conjugated vaccine; PCV13: 13-serotype pneumococcal conjugated vaccine; Ct: Cycle threshold.

## Competing interests

This work was supported in part by funds from the Pfizer Investigator-Initiated Research Grant Program. The authors declare that they have no competing interests related to this company beyond the funding received through the Investigator-Initiated Research Grant Program.

## Authors’ contributions

RS contributed to study design and drafted the primary manuscript. LH and IM performed laboratory work, data entry, and manuscript preparation. JMP and FC contributed to study design and manuscript preparation. All authors have read and approved the final manuscript.

## Pre-publication history

The pre-publication history for this paper can be accessed here:

http://www.biomedcentral.com/1471-2431/14/189/prepub

## Supplementary Material

Additional file 1: Table S1 *Streptococcus pneumoniae* real-time PCR molecular serotyping assays used with pleural fluid specimens. Click here for file
