# Supplementary material for: Direct Streptococcus pneumoniae real-time PCR serotyping from pediatric parapneumonic effusions
Source: BMC Pediatr. 2014 Jul 24;14:189. doi: 10.1186/1471-2431-14-189 (PMC4118202; doi:10.1186/1471-2431-14-189)
Supplement: Additional file 1: Table S1 — Streptococcus pneumoniae real-time PCR molecular serotyping assays used with pleural fluid specimens. [file 1471-2431-14-189-S1.docx]

| **S. pneumoniae Serotype** | **Gene** | **Forward Primer** | **Reverse Primer** | **Probe ***** | **Ref.** |
| --- | --- | --- | --- | --- | --- |
| Serotype 1 | *cpsa* | cgtgcggtaattgaagctatga | tgtggccccagcaactct | cttgcccttgtatagggt | 15 ** |
| Serotype 3 | *cpsa* | ggtcagcagaaagtatgcattgg | tcgtttatccagggtctgatga | tattggatgtggtttatcgtgaag | 15** |
| Serotype 4 | *wcij* | cggcaggcaaaccaattat | catctcgttcgggactaaca | caggagatgctaaaata | 16 |
| Serotype 5 | *cpsa* | ttacgggagtatcttatgtctttaatgg | cagcattccagtagcctaaaactaga | tctcagcaactctatttgg | 15** |
| Serotype 6A | *wcip* | gctagagatggttccttcagttgat | catactctagtgcaaactttgcaaaat | ctggctcatgatagtt | 16 |
| Serotype 6B | *wcip* | gctagagatggttccttcagttgat | catactctagtgcaaactttgcaaaat | actgtctcatgataatt | 16 |
| Serotype 7F /A | *wcwa* | aagcacagtgcgtgaacaat | aaaatctccctgtcccttcc | ctattccagaagaatctc | 16 |
| Serotype 9V/A | *cpsa* | tggaatgggcaaagggtagta | tcggttccccaagattttctc | ttaatcatgctaacggctcat | 15** |
| Serotype 14 | *cpsa* | cgactgaaatgtcactaggagaagat | aatacagtccatcaattactgcaatactc | attcgtttgccaatacttga | 15** |
| Serotype 18C/B | *gct* | ccctgaaactagttgggaaca | ttccaatcatcacccattaca | aaagtcagatgttaaagactac | 16 |
| Serotype 19A | *wcho* | gctgtgtttatgggggttgg | agagacgtttaggctcatttgc | atgcaaaatgctcacctag | 16* |
| Serotype 19F/B/C | *wcho* | aattcggtatttatgggagttgg | agagacgtttaggctcattagc | atgcaaaagtcaaatttaga | 16* |
| Serotype 23F | *wchv* | ctgggccaagatatttaaaagagagt | aattccgcatcagagtatgcaa | ttgctcttcgaaaaatgt | 16* |

**Additional File 1. *Streptococcus pneumoniae* real-time PCR molecular serotyping assays used with pleural fluid specimens**

** the published probe was modified as a minor grove binder was attached to probe; * modified from published by replacement of degenerate nucleotides with specific nucleotides to match serotype sequence. ***All assays were labeled with fluorescein amidite (FAM) and contained a minor grove binder.
